# The Brugada Syndrome: A Rare Arrhythmia Disorder with Complex Inheritance

**DOI:** 10.3389/fcvm.2016.00009

**Published:** 2016-04-25

**Authors:** Jean-Baptiste Gourraud, Julien Barc, Aurélie Thollet, Solena Le Scouarnec, Hervé Le Marec, Jean-Jacques Schott, Richard Redon, Vincent Probst

**Affiliations:** ^1^Service de Cardiologie, Centre Hospitalier Universitaire (CHU) de Nantes, l’institut du thorax, Nantes, France; ^2^Institut National de la Santé et de la Recherche Médicale (INSERM) Unité Mixte de Recherche (UMR) 1087, l’institut du thorax, Nantes, France; ^3^Centre National de la Recherche Scientifique (CNRS) UMR 6291, l’institut du thorax, Nantes, France; ^4^l’institut du thorax, Université de Nantes, Nantes, France

**Keywords:** Brugada syndrome, genetics, sudden death, cardiac arrhythmias, SCN5A

## Abstract

For the last 10 years, applying new sequencing technologies to thousands of whole exomes has revealed the high variability of the human genome. Extreme caution should thus be taken to avoid misinterpretation when associating rare genetic variants to disease susceptibility. The Brugada syndrome (BrS) is a rare inherited arrhythmia disease associated with high risk of sudden cardiac death in the young adult. Familial inheritance has long been described as Mendelian, with autosomal dominant mode of transmission and incomplete penetrance. However, all except 1 of the 23 genes previously associated with the disease have been identified through a candidate gene approach. To date, only rare coding variants in the *SCN5A* gene have been significantly associated with the syndrome. However, the genotype/phenotype studies conducted in families with *SCN5A* mutations illustrate the complex mode of inheritance of BrS. This genetic complexity has recently been confirmed by the identification of common polymorphic alleles strongly associated with disease risk. The implication of both rare and common variants in BrS susceptibility implies that one should first define a proper genetic model for BrS predisposition prior to applying molecular diagnosis. Although long remains the way to personalized medicine against BrS, the high phenotype variability encountered in familial forms of the disease may partly find an explanation into this specific genetic architecture.

## Introduction

The Brugada syndrome (BrS) is a rare inherited arrhythmia disease, first described in 1992, increasing the risk of ventricular fibrillation in apparently healthy young adults (1). It is suspected to be involved in 4–12% of cases of sudden cardiac death (SCD) in the general population and in at least 20% of SCD in patients with a structurally normal heart (1–3).

**Figure 1:**
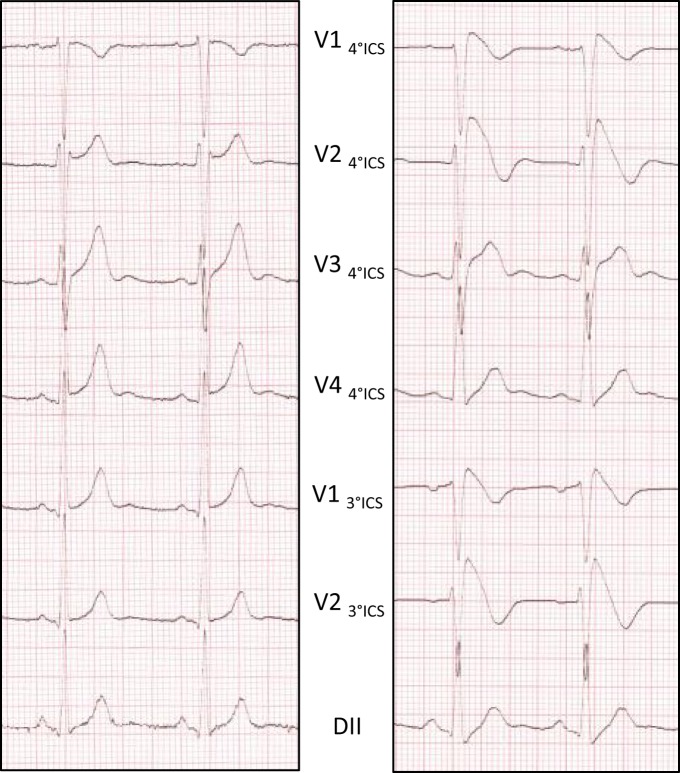
**Ajmaline testing reveals the Brugada ECG pattern**. ECG pattern is recorded at 1 mm/10 mV and 25 mm/s. Baseline ECG without aspect of BrS (left side). Type 1 Brugada pattern on the right precordial leads at the end of the test (right side).

Clinical diagnosis is based on a specific electrocardiographic (ECG) pattern defined in three consecutive consensus conferences (4–6). This ECG pattern, previously known as “type 1” ECG pattern, is defined as a ST segment elevation with a coved-type morphology ≥0.2 mV in one lead among the right precordial leads V1 and V2, positioned in the second, third, or fourth intercostal space occurring either spontaneously or after provocative drug test with intravenous administration of Class I antiarrhythmic drugs (6) (Figure 1). The ECG pattern may be transient in affected patients (7). To address this issue, unmasking drugs, such as ajmaline, flecainide, and procainamide, can be used to reveal this pattern (8), ajmaline showing higher sensitivity than flecainide and procainamide (4, 9, 10).

The high variability of the ECG pattern impairs proper assessment of its prevalence in the general population. Epidemiological studies have produced heterogeneous results regarding BrS incidence across the World. While estimated at 5 for 10,000 in western Europe and the USA, the prevalence of BrS seems higher in Southeast Asia, reaching 20 for 10,000 (11–13).

Aborted SCD is often the first symptom in BrS, with a mean age of 45 years at diagnosis and a four-time higher incidence in men than in women (14, 15). A third of the affected patients are identified after syncope, frequently preceded by vagal symptoms (14). The syncope could either be due to non-sustained VF or to a vaso-vagal episode without direct clinical relevance, rendering it hard for the practitioner to distinguish arrhythmic from ­non-arrhythmic etiology (16, 17). The majority of patients are asymptomatic at time of diagnosis. More than one-third of cases are identified during familial screening (14).

Implantation of a defibrillator is still the only efficient therapy in high-risk patients, with a 48% rate of appropriate device therapy at 10 years in patients with previous aborted sudden death. This rate falls to 12% among implanted asymptomatic patients, many affected patients remaining asymptomatic during all their life. Furthermore, device-related complications are frequent with a 30% risk at 10-year follow-up mainly due to lead dysfunction, inappropriate therapy, and infection (18, 19). These serious side effects in comparison to the very low arrhythmic risk for asymptomatic patients require accurate risk stratification and/or efficient drug therapy.

Only few clinical parameters allow risk stratification in BrS. The effectiveness of ventricular stimulation is still a matter of debate, and symptoms and spontaneous ECG pattern are still the two major parameters enabling risk stratification for SCD (6, 14, 20–23).

There is still need for medical therapies that could reduce arrhythmia occurrence and prevent SCD. Because successful trials were reported in limited series of patients, quinidine has been expected to be “the drug” for BrS. However, several recent studies failed to demonstrate its beneficial effects (6, 24–27).

There is accumulating evidence that implantable defibrillator is an effective and accurate therapy for symptomatic patients (18). Many clinical parameters have also been proposed for asymptomatic patients, but risk prediction in the latter group of patients remains particularly challenging because of the lack of reproducible and reliable data (28).

## Two Pathophysiological Models for BrS

Those unresolved questions concerning diagnosis and risk stratification for arrhythmia and therapy underlie the need for a better understanding of pathophysiological mechanisms in BrS. Two main pathophysiological hypotheses have been proposed to explain the ECG pattern.

Soon after the description of BrS, the first pathophysiological model was proposed, based on the existence of a transmural voltage gradient due to a repolarization heterogeneity across the ventricular wall (29, 30). According to this hypothesis, ST segment elevation could be due to either a loss of function of the sodium channel NaV1.5 responsible for the depolarization phase (phase 0 of the AP) favoring the expression of repolarization heterogeneity, an aggravation of this heterogeneity by a gain of function in one of the cardiac potassium channels responsible of the repolarization phases (phases 1 and 3 of the AP), or a loss of function of the CaV1.2 calcium channel that participate to the phase 2 of the AP (29).

This hypothesis has been matter to debate since the second hypothesis, based on a conduction delay in the right ventricular outflow tract, emerged from clinical observations (31–35). This conduction delay could be responsible for voltage gradients between RV and RVOT during and explain the BrS ECG pattern.

Twenty years of genetic research based on both technological and methodological progresses have started to depict the complexity of BrS pathophysiology (36, 37). This review aims to provide an integrated synopsis of those two decades of research and to suggest future directions for further genetic investigations against BrS.

## From a Familial Disease to the Identification of Rare Variants

**Figure 2:**
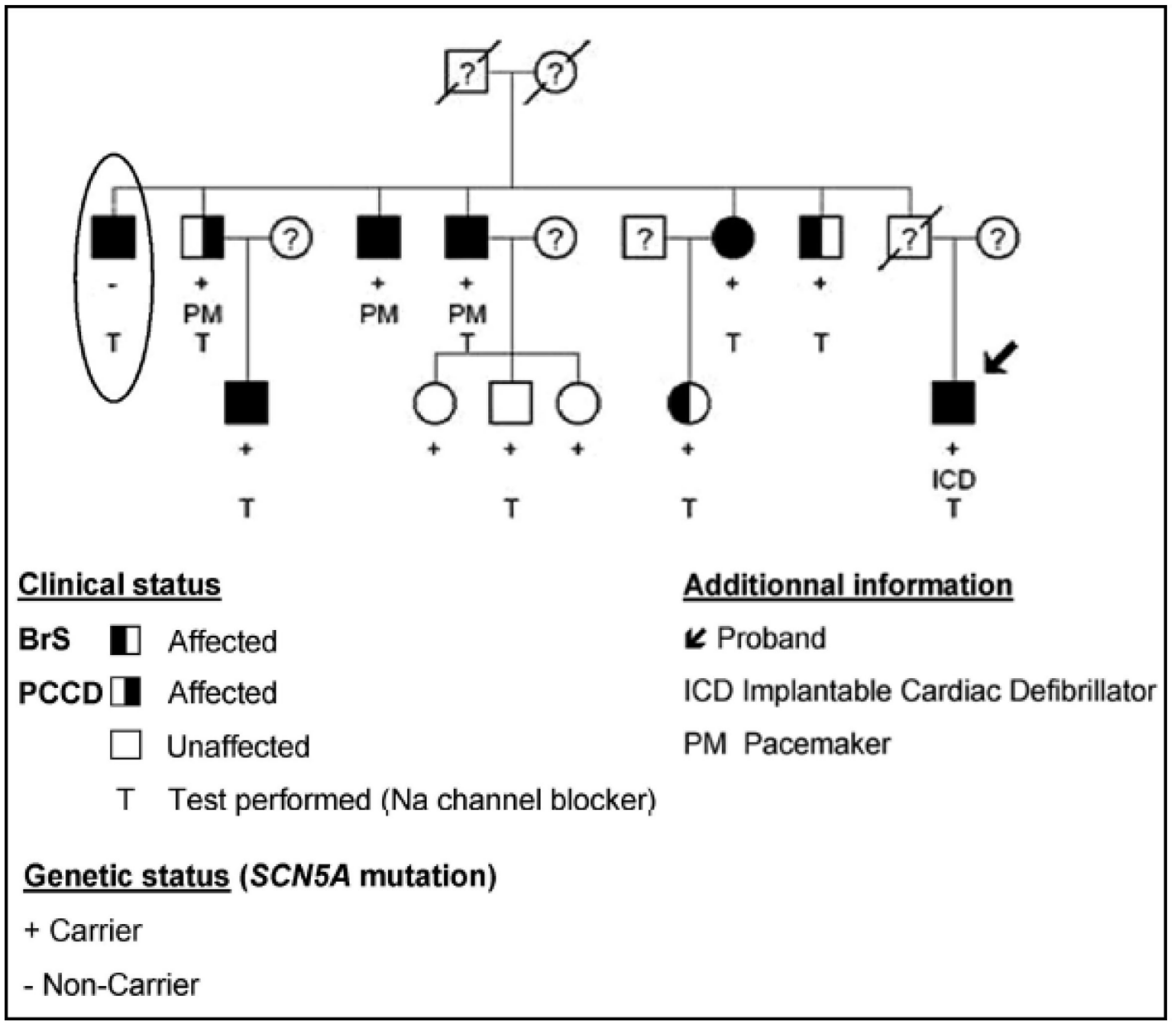
**The complex inheritance pattern of BrS**. Modified from Ref. (41). Incomplete penetrance of the *SCN5A* mutation is illustrated by the presence of unaffected carriers of the mutation (+). The patient highlighted by an ellipse presents with a BrS ECG aspect, despite the absence of the familial mutation. Affected family members carrying the *SCN5A* mutation present with progressive cardiac conduction disease (PCCD) (right half-filled symbol), BrS (left half-filled symbol), or both diseases (full-filled symbol). PCCD consists of right bundle branch block with PR interval lengthening and led to complete AVB in three patients, in whom a pacemaker (PM) was implanted.

With the initial report of two affected siblings, familial inheritance was suggested from the first description of the Brs in 1992 (1). Few years later, Kobayashi et al. described a two-generation family presenting with both SCD and persistent ST elevation in relatives (38), confirming the heritability of the disease. The genetic component of BrS was further demonstrated in several reports (39–41) (Figure 2). Today, familial history of SCD is reported for about 26% of affected patients. Additionally, 36% of affected patients are identified during familial screening after SCD or identification of BrS in the proband (14).

Brugada syndrome has been consistently reported as a monogenic disease with autosomal dominant mode of inheritance, caused by rare genetic variants with large effect size (1, 38). Loss-of-function mutations in the SCN5A-encoded α-subunit of the cardiac sodium channel (Nav1.5) were first identified in 1998 (42). Mutations in *SCN5A* are detected in 20–25% of cases, *SCN5A* appearing as the major susceptibility gene for BrS (43). More than 300 rare variants in *SCN5A* have been reported, while the contribution of other genes remains extremely low (43, 44). In a pediatric population affected by BrS, the prevalence of *SCN5A* mutations seems to be even higher (45).

In this context, genetics was initially expected to help the clinical management of patients with BrS. Although some *SCN5A* mutations – particularly those leading to premature truncation of Nav1.5 – have been reported as associated with higher arrhythmic risk, no such result has been further confirmed in randomized studies (14, 46–48).

Despite evidence for strong familial inheritance, familial linkage analyses on BrS have been largely unsuccessful. Only one gene, *GPD1L*, has been identified as a BrS-susceptibility gene using this approach (49). The causing mutation in *GPD1L* has been shown to affect Na^+^ channel trafficking to the plasma membrane, by modifying its oxydation state (49, 50). Every other gene reported so far has been identified through a candidate approach based on direct sequencing of genes with a known (or suspected) role in cardiac electrical activity.

So far, 23 genes have been related to BrS (Table 1). Based on pathophysiological hypotheses, those genes can be divided according to whether they affect the sodium current *I*_Na_ (*SCN5A*, *SCN10A*, *GPD1L*, *SCN1B*, *SCN3B*, *RANGRF*, *SCN2B*, *PKP2*, *SLMAP*, and *FGF12*), the potassium current *I*_K_ (*KCNJ8*, *KCNH2*, *KCNE3*, *KCND3*, *KCNE5*, *KCND2*, *SEMA3A*, and *ABCC9*), or the calcium current *I*_Ca_ (*CACNA1C*, *CACNB2B*, and *CACNA2D1*).

**Table 1:** **The 23 reported susceptibility genes for BrS**.

OMIM ranking	Gene	Protein	Prevalence in BrS cases	Functional effect of the mutation	Reference
BrS1	*SCN5A*	α subunit of the Nav1.5 sodium channel	20–25%	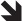 *I*_Na_	(42)
BrS2	*GPD1L*	Glycerol-3-phosphate dehydrogenase 1-like	Rare	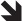 *I*_Na_	(49)
BrS3	*CACNA1C*	α subunit α1C of the Cav1.2 calcium channel	1–2%	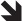 *I*_Ca-L_	(51)
BrS4	*CACNB2b*	β subunit Cavβ2b of calcium channel	1–2%	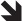 *I*_Ca-L_	(51)
BrS5	*SCN1b*	β subunit Navβ1 of sodium channel	Rare	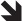 *I*_Na_	(52)
BrS6	*KCNE3*	β subunit MiRP2 of potassium channel	Rare	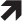 *I*_to_	(53)
BrS7	*SCN3b*	β subunit Navβ3 of sodium channel	Rare	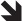 *I*_Na_	(54)
BrS8	*HCN4*	Hyperpolarization-activated cyclic nucleotide-gated channel 4	Rare	?	(55)
BrS9	*KCND3*	α subunit of the KV4.3 potassium channel	Rare	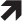 *I*_to_	(56)
BrS10	*KCNJ8*	α subunit of the KIR6.1 potassium channel	Rare	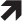 *I*_KATP_	(57)
BrS11	*CACNA2D1*	δ subunit Cavα2δ1 of calcium channel	Rare	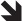 *I*_Ca-L_	(58)
BrS12	*KCNE5*	β subunit of potassium channel	Rare	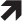 *I*_to_	(59)
BrS13	*RANGRF*	RAN guanine nucleotide release factor	Rare	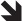 *I*_Na_	(60)
BrS14	*KCND2*	α subunit of the KV4.2 potassium channel	Rare	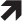 *I*_to_	(61)
BrS15	*TRPM4*	Calcium-activated non-selective ion channel	Rare	?	(62)
BrS16	*SCN2B*	β subunit Navβ2 of sodium channel	Rare	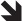 *I*_Na_	(63)
BrS17	*PKP2*	Plakophilin 2	Rare	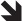 *I*_Na_	(64)
BrS18	*ABCC9*	ATP-sensitive potassium channels	Rare	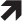 *I*_KATP_	(65)
BrS19	*SLMAP*	Sarcolemma-associated protein	Rare	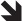 *I*_Na_	(66)
BrS20	*KCNH2*	α subunit of the HERG potassium channel	Rare	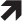 *I*_Kr_	(67)
BrS21	*SCN10A*	α subunit of the Nav1.8 sodium channel	<5%	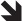 *I*_Na_	(68, 69)
BrS22	*FGF12*	Fibroblast growth factor 12	Rare	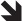 *I*_Na_	(70)
BrS23	*SEMA3A*	Semaphorin family protein	Rare	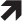 *I*_to_	(71)

*Functionnal effect on current are described with arrow, except for HCN4 mutation for which it remain unclear (?)*.

## Limits in Interpreting Rare Variants Carried by Patients with BrS

In the last decade, the emergence of massively parallel sequencing [or next-generation sequencing (NGS)] has considerably facilitated genetic screening and reduced its cost (72–76). Combined to the availability of the reference assembly of the human genome (77, 78), NGS-based approaches have revealed the high variability of the human genome, with at least 300–600 functional genetic variants detected in each exome (i.e., the whole coding portion of a single genome) (75) – and has retrospectively changed the interpretation of previous rare variants identified by candidate gene approach. The investigation of large number of exomes revealed the extraordinary prevalence of rare variants among each individual. As an illustration, the sequencing of 60,706 exomes identified about 7,500,000 variants from which 99% have a frequency of <1% (http://biorxiv.org/content/early/2015/10/30/030338).

Extreme caution should thus be taken when interpreting the rare genetic variants detected among patients with BrS, since the clinical implication of finding those variants remains doubtful in the absence of statistical association and/or of evidence supporting a functional effect in relation with cardiac electrical activity (79–82).

Furthermore, a recent study has illustrated the weakness of candidate approaches on small pedigrees, by highlighting the high frequency of some genetic variants previously associated with BrS among 6,500 individual exomes from the Exome Sequencing Project (83). One variant in particular, which was related to BrS based on functional evidence, showed a minor allele frequency of 4.4% among the 6,500 individuals. This result was confirmed in a healthy Danish control population, suggesting that a proportion of the genetic variants reported as causing BrS are actually not pathogenic. Interestingly, 93% of the *SCN5A* variants reported as causing BrS are not present among the control population, thus reinforcing the pivotal role of this gene.

**Figure 3:**
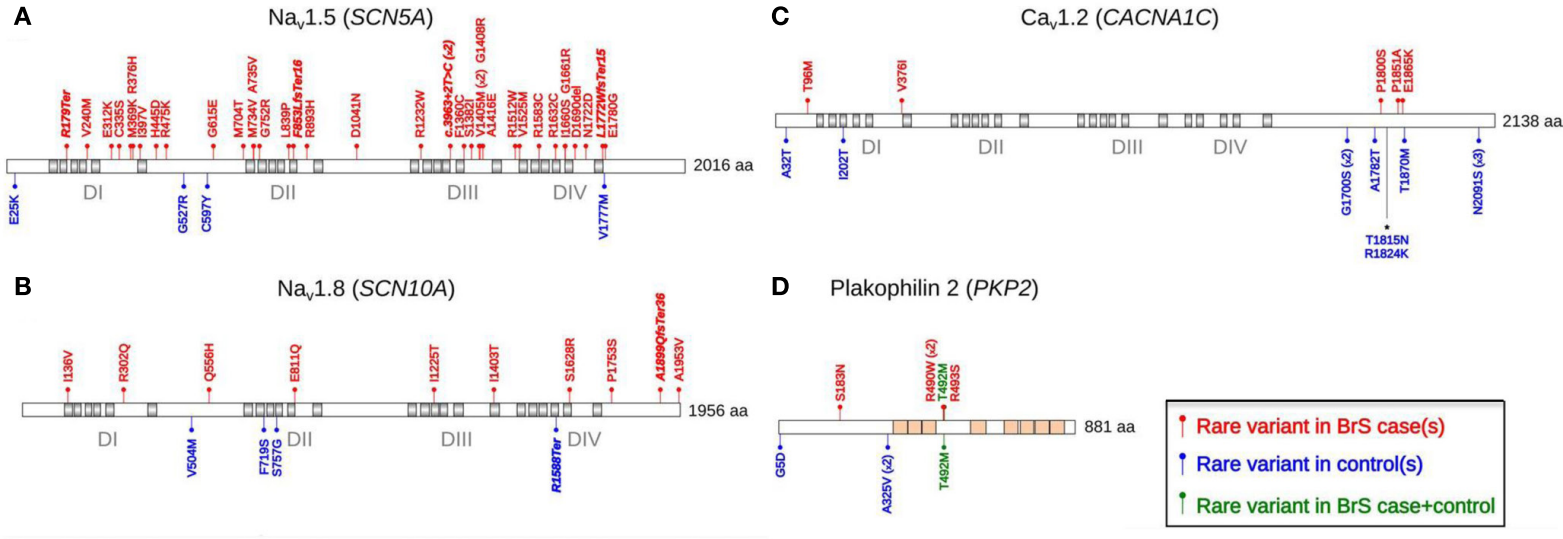
**The distribution of rare coding variants detected across four selected arrhythmia-susceptibility genes among 167 BrS cases and 167 healthy individuals**. Modified from Ref. (44). *SCN5A* **(A)**, *SCN10A* **(B)**, *CACNA1C* **(C)**, and *PKP2* **(D)** are the four genes exhibiting the largest numbers of rare coding variants among BrS cases. Rare coding variants (minor allele frequency <0.1%) are represented in red (cases) and blue (controls). Green variants are detected in both cases and controls.

By testing the burden of rare coding variants in 45 arrhythmia-susceptibility genes among 167 BrS cases versus 167 control individuals, we have also recently demonstrated the limitation of previous candidate approaches (44). Indeed, for every tested gene except *SCN5A*, rare variants were found in the same proportion in cases than in controls. Figure 3 shows the distribution of rare variants among cases and controls for the protein products of four genes: *SCN5A*, *SCN10A*, *CACNA1C*, and *PKP2*. The distribution of rare variants across the functional domains of the *CACNA1C* product indicates that the C-terminal tail, which was previously considered as pathogenic in BrS, may in fact be highly polymorphic. On the opposite, most rare variants detected along the protein encoded by *PKP2* among BrS patients reside in a small interval coding for four amino acids. The *PKP2* gene has been previously associated with BrS by decreasing functional Na channel expression through modification of microtubule anchoring (64). The small *PKP2* interval emphasized in this study may be a preferential site of such interaction.

Rare genetic variants appear more evenly distributed across *SCN10A* and less predictive of any potential pathophysiological mechanism. In fact, the functional effects of these rare variants affecting *SCN10A* are largely debated. *SCN10A* gene, which encodes the sodium channel Na_v_1.8, was initially described in neurons physiology (84, 85). Further investigations illustrated a potential role in cardiac electrophysiology, particulary as a modulator of cardiac conduction (86, 87). Recently, Hu et al. described rare variants in the *SCN10A* gene, in 16.7% of 150 patients affected with BrS (68). Furthermore, they demonstrated that the *SCN10A* variants R1268Q and R14L reduced cardiac sodium currents (68). However, although relevant biological effects are reported for some variants, most variants are also reported in control populations. Behr et al. have recently underlined this issue (69). Using an extended control population, they decreased the yield of such variants from 16.7% in the Hu et al.’s study to 5.1% in a different set of BrS probands (68). Additionally, only two over seven familial pedigrees available with such variants demonstrated segregation with the BrS.

Coding genetic variants in candidate genes are usually classified as likely pathogenic if they are extremely rare or absent from control populations. However, private genetic variants are found in control populations, and many rare variants predicted as damaging are carried by apparently healthy individuals (44, 83). As an example, in the *SCN5A* gene, rare functional variants can be found in about 2% of control patients and even in 5% in non-white population (88). Thus, considering *SCN5A*-mediated BrS account for about 20% of cases and that background noise of rare variant with minor allele frequency under 1/10,000 is approximately 2%, there is a 10/1 signal to noise ratio that means a 10% risk of false positive in possibly damaging rare *SCN5A* variants (82). As prevalence of asymptomatic BrS in the general population is unknown, this percentage may be over estimated. However, as BrS is a rare disease, the proportion of false positive variants remains, in any case, too high to be confident with a direct translation of new rare variants in clinical practice.

On the opposite, some rare variants detected among BrS patients are reported as benign by prediction algorithms though they modify the function of the protein. As an example, while one *SCN3B* variant has been associated with BrS and reported as impacting the sodium current density, it is considered as benign by prediction algorithms such as SIFT and PolyPhen-2 (54, 89, 90). This demonstrates the strong limitations of such prediction algorithms and the need for functional studies and/or segregation analyses to better assess the causality of rare variants.

From that perspective, mutations in L-type calcium channels (*CACNA1C*, *CACNB2B*, and *CACNA2D1*) that were considered as associated with about 4% of BrS cases are of particular interest (43). The L-type calcium current *I*_Ca-L_ is a perfect candidate to explain BrS physiopathology, due to its central role in action potential dome (phases 2 and 3) and in the “depolarization” hypothesis (91). However, functional studies on mutations in L-type calcium channels are scarce in the literature. Moreover, mutations in *CACNA1C* among BrS cases and controls are mostly located within the C-terminal tail of Ca_v_1.2, thus suggesting a high genetic variability of the domain (Figure 3). Although *CACNA1C* mutations seem to play lesser role than previously reported, this particular gene remains involved in a small subset of BrS cases, in particularly those with combined phenotypes of BrS and short QT syndrome (92).

These accumulated data demonstrate that in order to avoid misinterpretation of genetic variants: (1) functional prediction algorithms should be used cautiously and (2) ancestry-matched control populations should be systematically considered. Furthermore, familial segregation analysis and/or extended functional testing are mandatory before associating rare coding variants to disease susceptibility.

Following these guidelines, no previously reported susceptibility gene except *SCN5A* seems to contribute significantly to BrS pathophysiology. Although *SCN5A* remains the major gene involved in BrS with about 20% of carriers among probands (43, 44), a proportion of rare variants residing in this gene – particularly among those of uncertain functional effect – could play no role in relation with the disease (82).

## The Complex Inheritance of BrS: Toward a New Genetic Model

Since the discovery of *SCN5A* as the first susceptibility gene for BrS, this disorder has been consistently reported as a monogenic disease with autosomal dominant mode of inheritance, caused by rare genetic variants with large effect size (1, 38, 42). *SCN5A* remains the only major susceptibility gene for BrS, with more than 300 coding variants described among more than 75% of the genetically diagnosed patients (43, 93). However, hardly any of the large family pedigrees with BrS provides evidence for Mendelian inheritance. Most familial forms indicate a genetic model with incomplete penetrance and remain genetically undiagnosed.

We have investigated the cosegregation of *SCN5A* mutations with BrS among large genotyped families (41). *SCN5A* mutations exhibit low penetrance (61% after drug testing) in families, leading to poor genotype/phenotype correlations. More surprisingly, among five pedigrees, we could identify eight affected members who did not carry the familial *SCN5A* mutation (Figure 2). This lack of genotype/phenotype correlation is further emphasized in other families with variable cardiac phenotypes associated with a same *SCN5A* mutation. Indeed, although a Na current decrease could lead to cardiac conduction or sinus node dysfunction, the description of relatives sharing the same *SCN5A* mutation but presenting with either BrS or a progressive cardiac conduction disease question about the relevance of a monogenic model (94, 95). A similar issue involving *SCN5A* mutation has been described with BrS and long QT syndrome (96).

**Figure 4:**
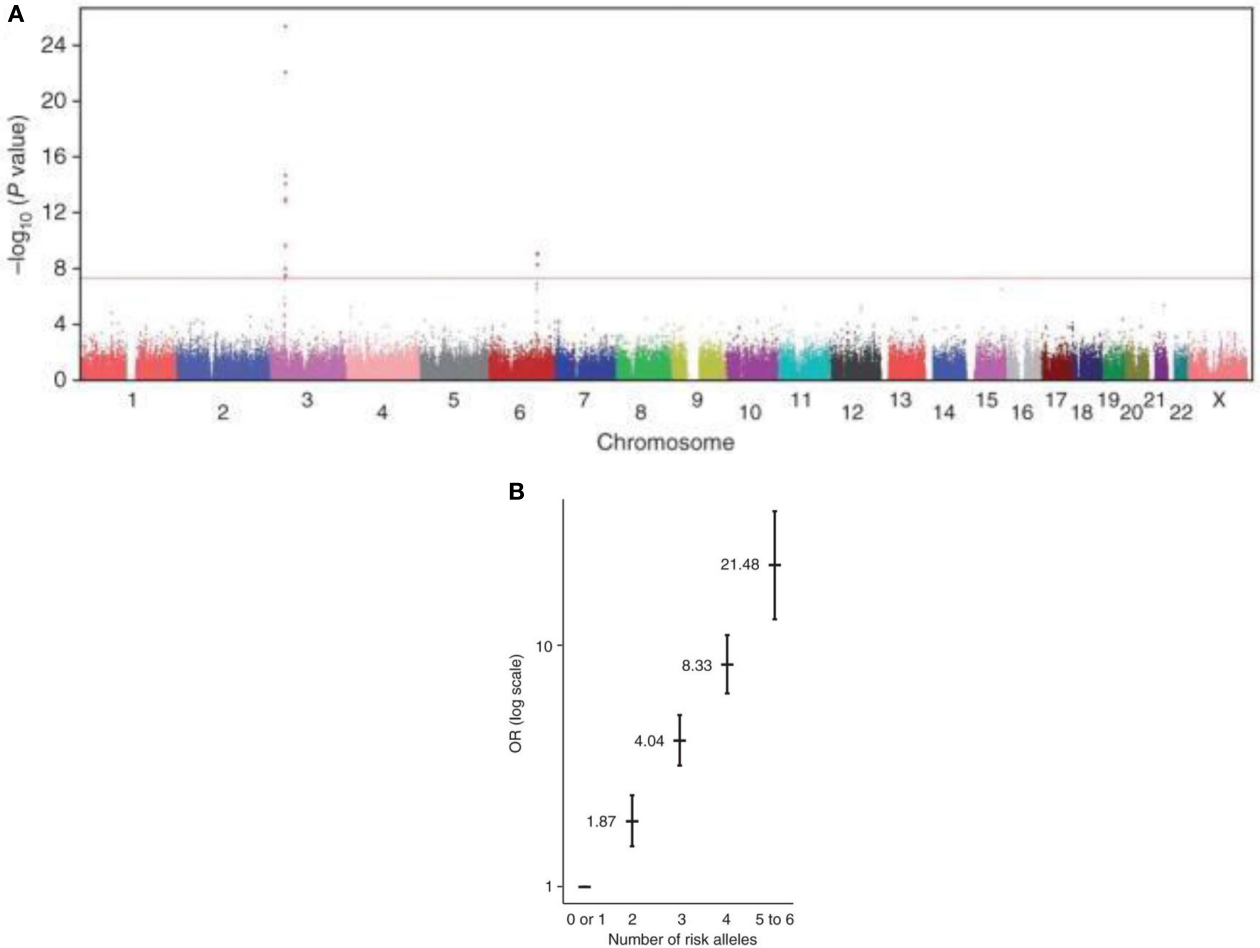
**A genome-wide association study on BrS**. Modified from Ref. (101). **(A)** Manhattan plot revealing signal associations between two SNP (SCN10A and HEY2) and BrS. Statistical significance is represented with a red line (*P* = 5 × 10^−8^). A third haplotype of SCN5A reached genome-wide significance after replication analysis. **(B)** The cumulative effect of the six common risk alleles on susceptibility to BrS. Odds Ratio (OR) is plotted on the vertical axis and the cumulative number of alleles (from 0 to 6) in horizontal axis.

These observations have led us to seek for genetic factors modulating the risk of Brugada ECG phenotype. To explore the potential role of common genetic variants in susceptibility to Brs, we have recently coordinated an international genome-wide association study (GWAS) on BrS. By comparing allele frequencies of common haplotypes genome wide among 312 index cases versus 1,115 control individuals, we identified three loci associated with susceptibility to BrS (Figure 4A). The three hits were then replicated on independent case–control sets from Europe and Japan. We found that their cumulative effect on disease susceptibility was unexpectedly large, with an estimated odds ratio of 21.5 in the presence of more than four risk alleles versus less than two (Figure 4B). This study demonstrates that an aggregation of genetic polymorphisms can strongly influence the susceptibility to BrS and confirms that the mode of inheritance for this arrhythmia disorder is far more complex than previously described.

Two association signals reside at the *SCN5A-SCN10A* locus. Both common risk alleles have previously been associated with cardiac conduction traits in the general population (97). This finding demonstrates that genetic polymorphisms modulating cardiac conduction can also influence susceptibility to cardiac arrhythmia. One haplotype is located inside the *SCN10A* gene, of which involvement in the pathophysiology of BrS is still matter to debate. van den Boogaard et al. provided evidence that the *SCN10A* haplotype contain was an enhancer region for both *SCN10A* and *SCN5A* genes (98). They further demonstrated that a common variant (rs6801957) of this locus, associated with cardiac conduction trait and in high linkage disequilibrium with rs10428132, alters a transcription factor binding site for *TBX3*/*TBX5* and reduces the *SCN5A* expression (99). This may explain the high phenotype variability observed in BrS patients even within a same family.

The third association signal resides near the *Hey2* gene, which encodes a basic helix-loop-helix transcriptional repressor expressed in the cardiovascular system. The implication of this gene in susceptibility to BrS was previously unknown (100). Interestingly, Hey2 presents a gradient of expression across the ventricular wall in mirror image with *SCN5A* expression suggesting a possible (indirect) regulation mechanism. Despite no ECG changes, Hey2 heterozygous knockout mice (Hey2^+/−^) present interesting findings for BrS pathophysiology. Conduction velocity seems specifically increase in the right outflow tract in which cellular action potential present both increase in AP upstroke velocity and repolarization (101). These data uncovered the role of Hey2 in the cardiac electrical function and more specifically in the pathogenesis of BrS. Among its role on BrS phenotype, common variant in this gene could also presented with a protective role from ventricular fibrillation in BrS patients by regulating the repolarization current (102).

## Conclusion

Almost two decades ago, the first description of a mutation in *SCN5A* gene has paved the way of genetics in BrS. As BrS was initially described as a Mendelian disease with low penetrance, many studies have been performed to track genetic variants in families affected by this syndrome. However, in most cases, studies were unable to show positive linkage. In a very large majority of cases, putative causing genes were identified through a “candidate gene approach” based on pathophysiological hypotheses. In these *a priori* approaches, the results were “validated” by the rarity of the genetic variants identified, while aberrant linkage results were “explained” by non-penetrance or phenocopies.

In the recent years, NGS technologies have dramatically expanded our capacity to sequence genomes. It has also revealed the high variability of the human genome, underlying the extreme caution that should be taken to avoid misinterpretation of the potential association of rare variants with BrS. Thus, recent burden tests have questioned the implication of several genes previously identified as there distribution was similar in the normal population and affected patients. For now, only rare variants in *SCN5A* gene seem to be significantly associated with the syndrome.

However, genotype/phenotype studies among BrS families with *SCN5A* mutation carriers have highlighted a complex mode of inheritance for this syndrome. In line with these reports, a GWAS has recently identified three common risk haplotypes for the Brugada ECG pattern.

It is now established that the molecular mechanisms leading to BrS involve both rare and common genetic variants, underlying the need for better understanding the genetic architecture of BrS prior to applying genetics as a diagnostic tool. For the next future, one of the challenges that could contribute to a more efficient strategy for BrS would be to decipher the role of the combination of variants both for diagnosis and prognosis.

Another source of progress regarding risk stratification among BrS patients could go through the identification of specific ECG indices associated with higher risk of (fatal) arrhythmia. Genetic variants at the *SCN5A*, *HEY2*, and *SCN10A* loci have been associated with arrhythmia occurrence in independent studies (47, 102, 103). Integrating such effects toward establishing a global genetic model for BrS is the next step before including genetic testing into the clinical management of BrS.

Besides the direct benefit of this research on the BrS for itself, it appears increasingly that this primary electrical disorder affecting the young adult (with no identifiable structural abnormalities and presenting limited exposure to environment side effect) may represent a relevant model for the identification of markers and mechanism implied into broader common cardiac arrhythmias. Retrospectively, *SCN10A* common variant identified in the BrS GWAS study have been also associated with the risk of VF in the context of myocardial infarction and with the pacemaker implantation rate (103, 104). Additionally, a protective role against developing AF has been suggested for both common variants previously identified as risk alleles for BrS at the SCN10A–SCN5A locus. This reinforces the interest of rare diseases to help identifying the pathophysiological bases of common pathologies. As they constitute homogenous groups of patients, rare arrhythmia disorders can provide new molecular insights that may be relevant to the broader health issue of SCD (105).

## Author Contributions

All authors authored sections of the manuscript, contributed to the figure design, and approved the final version.

## Conflict of Interest Statement

The authors declare that the research was conducted in the absence of any commercial or financial relationships that could be construed as a potential conflict of interest.
